# Socio-demographic variables among a group of patients with cognitive decline

**DOI:** 10.3389/fpsyg.2026.1770849

**Published:** 2026-03-19

**Authors:** Febronia Riggio, Palmira Faraci, Massimo Raffaele, Chiara Rizzotto, Amelia Gangemi

**Affiliations:** 1 Department of Cognitive Sciences (COSPECS), University of Messina, Messina, Italy; 2 Department of Human and Social Sciences, Kore University of Enna, Enna, Italy; 3 Department of Clinical and Experimental Medicine, University of Messina, Messina, Italy

**Keywords:** age, cognitive decline, domicile, education, gender, marital status, occupation, socio-demographic variables

## Abstract

**Objective:**

The present study aims to explore the relationship between socio-demographic characteristics and cognitive decline.

**Method:**

The sample included 124 participants (58.87% females), aged 62 to 91 years (M = 75.28, SD = 8.07), who were divided into two groups based on cutoff scores from the Mini-Mental State Examination (MMSE) and the Addenbrooke’s Cognitive Examination-III (ACE-III): 41 individuals were classified as cognitively healthy, and 83 were identified as suffering from cognitive decline. A series of standard multiple regression analyses were performed to predict cognitive performance based on gender, age, education level, work, domicile, and marital status.

**Results:**

The results showed that socio-demographic variables (i.e., age, education level, living in urban areas, and less cognitively demanding professions) were associated with cognitive domains (attention, memory, language, fluency and visuo-spatial ability).

**Conclusion:**

Sociodemographic variables have been demonstrated to be associated with cognitive performance. Our findings may prove useful in improving the diagnostic profile of individuals with cognitive decline. Using sociodemographic characteristics to identify individuals at risk might support the development of targeted interventions, improving quality of life and reducing associated straining factors.

## 1 Introduction

Dementia is a comprehensive term referring to a group of neurological disorders characterized by a cognitive decline severe enough to interfere with activities of daily living. Clinical risk factors of this neurological condition include age, family history, cardiovascular risk factors, smoking, excessive alcohol consumption, and low educational level (risk of disease decreases by approximately 17% for each year of education) (Evans et al., 1997; Livingston et al., 2020).

According to the World Health Organization (WHO), dementia is a global public health priority, with estimates suggesting that approximately 50 million people worldwide are currently affected by dementia and that this number is predicted to increase by 2050 (World Health Organization, 2021). In Europe, the number of cases per year is expected to double (Georges et al., 2020). The prevalence of dementia differs significantly across regions and countries. Several studies have been conducted to estimate prevalence rates, and the results show significant variations. A meta-analysis published in The Lancet Neurology in 2020 (Livingston et al., 2020) reported that the number of people with dementia is much higher in low- and middle-income countries due to larger populations and higher longevity rates. Also, Steptoe and Zaninotto (2020) analyzed the long-term health of aging conditions associated with socioeconomic status in a sample of 5,000 English people. Their findings indicated that more disadvantaged economic conditions were associated with greater negative changes in participants’ physical, cognitive, and emotional abilities. Even when other clinical variables, such as smoking or marital status, were included, the negative effects persisted. These results demonstrate that economic status and social conditions are determinants for the quality of aging. With regard to risk factors, few studies have focused on socio-demographic variables. A number of factors, including gender, age, and education, have been identified as significantly impacting cognitive performance and the likelihood of developing dementia. Integrating these variables with the results of a global cognitive function assessment could improve the prediction of dementia.

The aim of this study is to investigate the socio-demographic characteristics of patients with cognitive decline, which may serve as an additional step for early diagnosis.

### 1.1 Socio-demographic variables

- Female gender

Female gender is an important risk factor for cognitive decline; women showing a higher risk of developing conditions such as Alzheimer’s disease and other forms of dementia compared to men. There are several hypotheses that seek to explain this gender disparity in cognitive decline. In women older than 75 years, rates of hypertension, dyslipidemia, and diabetes are higher than in men of similar age. Apolipoprotein *ε* 4 genotype status appears to be prevalent and influences memory performance in women compared with men (Azad et al., 2007). Moreover, the study published in The Lancet Healthy Longevity (Chen et al., 2021), investigating gender differences in the aging, shows that older women generally have a longer lifespan than men, on average 4.5 years longer. However, older women are more susceptible to chronic diseases and disabilities, resulting in a poorer quality of life. They are more likely to develop diseases, as they often outlive their spouses. In addition, social isolation may impose an additional mental and cognitive burden on older women.

An additional hypothesis relates to low education in women referred to socio-cultural reasons; in the first half of the 20th century, the lower years of education in women than in men, could pose a gender’s difference about high risk for Alzheimer’s disease and other forms of dementia in women (Mielke et al., 2014).

In addition, recent research has shown that women are more exposed to hormonal changes during their life cycle stages, such as menopause. Observational studies have focused on the role of estrogen and its impact on cognition. In particular, the role of reduced estrogen levels in post-menopause has been linked to an increased risk of developing dementia. However, a causal relationship has not been established, as studies have also considered other potential risk factors (Pertesi et al., 2019; Basit et al., 2023).

- Age

Age is a major risk factor for cognitive decline. With advancing age, the brain undergoes natural degeneration processes that can affect cognitive function (Marra et al., 2011). This degeneration may involve brain atrophy, decreased neuronal connections, and loss of white matter. In addition, with advancing age, characteristic of Alzheimer’s disease, as the mechanisms of amyloid beta protein accumulation and neurofibrillary tau protein tangles, are triggered (Fjell and Walhovd, 2010; Damoiseaux, 2017). Aging is frequently associated with an increased prevalence of chronic diseases such as diabetes, hypertension, and cardiovascular disease, which can adversely affect brain health and the risk of cognitive decline (Fayosse et al., 2020).

- Education and the relationship with cognitive reserve

Years of education have been found to be a determining factor in building cognitive reserve, which, in turn, plays a crucial role in delaying cognitive decline. Cognitive reserve refers to the brain’s ability to tolerate structural or functional damage, maintaining the efficiency of cognitive functions (Stern, 2002). Years of education can directly influence cognitive reserve, providing a foundation of knowledge and cognitive skills that contribute to the preservation of brain function over time, concerning “*life-long learning*” (Corbo et al., 2023). A recent study (Gonzalez-Gomez et al., 2024) investigated the influence of education on 1.412 patients from Latin America (LA) and the USA. Disparities in education between Latin Americans and the USA correlated with brain morphological changes, especially in patients from LA countries. Reduce gray matter volume and lower functional connectivity of brain areas are resulted in LA than in USA population, increasing the risk of cognitive decline. Education emerged as a critical factor in the classification of aging and dementia across geographical regions and etiological subtype of dementia, e.g., Alzheimer’s Disease and Fronto-Temporal degeneration.

- Occupation

A systematic review and meta-analysis (Huang et al., 2020) showed the impact of occupational type on the development of dementia, suggesting that mental or manual work can influence the degree of exposure to dementia. According to International Standard Classification of Occupations (ISCO), mental work (or stimulating occupations, such as intellectual jobs, e.g., scientists, academic roles, etc.) (ISCO definition) (Ganzeboom, 2010) is associated with enhanced cognitive functioning in aging (Bonaiuto et al., 1995; Bosma et al., 2003; Smart et al., 2014; Then et al., 2017). Individuals engaged in manual job are often subject to occupational and environmental risk such as air pollutants, chemical agents, and fine particulate matte that have been associated with a increased risk of cognitive decline (Belsky and Baccarelli, 2023).

Rodriguez et al. (2021) showed that if individuals have engaged in mental work, allele e4 of the gene APOE, associated with the beginning of dementia, has less impact on the development and rapidity of cognitive decline. This hypothesis could be supported by the protective factor of “cognitive reserve” (Stern, 2002) as continued and modifiable mental stimulation can act as a protective factor against cognitive impairment (Amanollahi et al., 2021; Khalaila et al., 2024). Although intellectual work is generally regarded as a protective factor against pathological aging, the systematic review by Bufano et al. (2024) highlights that even those engaged in mentally demanding occupations, may experience negative effects on the physical dimension. These include cognitive impairments associated with stress, sleep disturbances and sedentary behavior.

Psychosocial workplace factors was also studied (Seidler et al., 2004). Authors studied the relationship between these factors and dementia. Results showed that challenge and control possibilities at work are such as protective factors against dementia; working under conditions of high perceived risk of making errors has been associated within increased likelihood of being diagnosed with dementia, because of chronic stress and diseases such as anxiety.

- Marital status

A systematic review and a meta-analysis (Sommerlad et al., 2018) that included 812.047 people worldwide found that the risk of dementia risk is elevated in lifelong single people (+42%) and in widowed people (+20%), because the end of a marriage due to mourning may directly increase the risk of dementia due to stress. No significant differences were found for divorced people.

The limitation identified in the studies about marital status is the lack of registration of single individuals in outpatient clinics. Indeed, partners often recognize the cognitive impairment of the patient, suggesting medical checkups. Single individuals may be slower to become aware of their cognitive deficits (Liu et al., 2020).

- Is the quality of aging related to the environment?

The environment affects human aging and quality of life. A recent review (Plagg and Zerbe, 2021) showed that public health depends on the natural characteristics of the habitat, such as air, climate, drinking water quality, and soil. These dimensions are also involved in the health of the elderly (Cassarino and Setti, 2015). Indeed, the quality of the environment in which one lives, such as noisy or heavily polluted environments, promotes the development of age-related diseases, which in turn are associated with the social and economic status of the individual (Belsky and Baccarelli, 2023).

The aim of this study is to explore how sociodemographic characteristics and domain-specific cognitive performance are distributed among individuals with and without cognitive decline, and to examine their role of predictors with cognitive scores.

## 2 Method

- Participants

The sample was enrolled at a Department of Neurology in Messina, from January to December 2023. The final sample comprised 124 participants (73 females and 51 males). Group allocation was based on age and education adjusted MMSE cut-off scores, resulting in 41 individuals classified as cognitively healthy and 83 individuals classified as experiencing cognitive decline. All cognitive assessments were conducted under the supervision of a neurologist. The ACE-III was administered to all participants but was used solely as a continuous measure of cognitive performance rather than for diagnostic grouping. Concerning the age variable, the sample was divided into years younger and older than 75; this range was chosen based on the WHO classification of age groups (age 75 and above is considered old age). For the education variable, 8 years of schooling was considered as the threshold; the sample was divided into two groups: those with higher education (high school diploma, bachelor’s degree) and the group of those with less than or equal to eighth grade education, a frequent condition due to the culture of the area (Brunetti, 2021), considering the average age of the sample. To investigate the residence variable, the sample was divided into those who live in the city and those who live in the province or smaller islands.

- Tools and procedures

Socio-demographic and clinical data were collected. Data were entered into a Microsoft Excel database. The Addenbrooke’s Cognitive Examination -III (ACE-III) and the Mini-Mental State Examination (MMSE) were used as neuropsychological tests. The time for administration was approximately 40 min. Items common to the MMSE and ACE-III were administered a single time. Before administration, subjects were informed about the purpose of the research. Following, they were asked to sign the informed consent.

The procedures of the present study have been approved by the Ethics Committee of Department COSPECS, University of Messina.

- Addenbrooke’s Cognitive Examination III (ACE-III)

The Addenbrooke’s Cognitive Examination-III (ACE-III) (Pigliautile et al., 2019) is a neuropsychological assessment tool used to evaluate global cognitive functioning. Cognitive Domains assessed are attention and spatio-temporal orientation, memory, phonological and categorical fluency, language, and visuospatial skills. The maximum obtainable score is 100 points. The test takes approximately 30 min to administer.

- Mini-Mental State Examination (MMSE)

The Mini-Mental State Examination (MMSE) (Measso et al., 1993) is a widely used rapid assessment of global cognitive functioning, particularly spatial and temporal orientation, memory, attention, language, and visuospatial skills. The test includes questions and tasks that assess several cognitive areas, such as temporal and spatial orientation, short- and long-term memory, computational ability, language ability, and visuospatial ability.

The maximum score obtainable is 30 points, with lower scores indicating possible cognitive deficits. Scores are interpreted and adjusted for the individual’s age and education.

### 2.1 Data analyses

Statistical analyses were performed using Jamovi and R (The Jamovi Project, 2025; Team, 2023).

A series of standard multiple regression analyses were conducted in order to ascertain the extent to which socio-demographic characteristics predicted performance across the cognitive domains assessed. A series of regression models were estimated for each cognitive outcome, including attention, memory, fluency, language and visuospatial abilities, as well as for their respective subcomponents. The socio-demographic predictors entered into each model were gender, age group (≤75 vs. >75 years), education level, marital status (married or unmarried), working status, and domicile. For each cognitive domain and subtest, the full model was subjected to a statistical significance test, followed by an examination of individual regression coefficients (*β*), associated t values, *p-*values, and partial correlations to ascertain the strength and direction of each predictor’s unique effect. The degree of fit of the model was measured using the coefficient of determination (*R*^2^), which provided an estimate of the proportion of the variance in cognitive performance that could be explained by the socio-demographic variables. This analytic approach facilitated a comprehensive evaluation of the impact of demographic characteristics on variability in cognitive functioning across multiple domains.

## 3 Results

In accordance with the study aims and to ensure a coherent analytical framework, we defined the five ACE-III domain total scores (Attention & Orientation, Memory, Fluency, Language, and Visuospatial abilities) were considered primary outcomes. The statistical analyses and tables were organized to reflect this distinction, with domain-level results (Table 1) and subtest-level analyses (Table 2) reported in the main manuscript.

**Table 1:** Multiple regression of total scores of ACE-III’ domains.

Variables	Attention’s total score			
	*β*	*t*	*p*	Partial correlation
Gender	−0.07	−0.40	0.689	−0.037
Over75	−0.02	−0.14	0.887	−0.013
Education level	0.36	3.22	0.001**	0.285
Work	−0.06	−0.60	0.548	−0.055
Domicile	−0.19	−0.99	0.321	−0.091
Marital status	0.18	0.89	0.371	0.082

Variables	Memory’s total score			
	*β*	*t*	*p*	Partial correlation
Gender	−0.01	−0.07	0.942	−0.006
Over75	−0.25	−1.46	0.145	−0.134
Education level	0.44	4.12	<0.001***	0.356
Work	−0.01	−0.15	0.879	−0.014
Domicile	−0.13	−0.74	0.458	−0.068
Marital status	0.12	0.64	0.519	0.059

Variables	Fluency’s total score			
	*β*	*t*	*p*	Partial correlation
Gender	−0.02	−0.17	0.863	−0.015
Over75	−0.11	−0.68	0.495	−0.063
Education level	0.37	3.53	<0.001***	0.310
Work	0.15	1.50	0.1351	0.137
Domicile	−0.11	−0.63	0.527	−0.058
Marital status	0.02	0.12	0.901	0.011

Variables	Language’s total score			
	*β*	*t*	*p*	Partial correlation
Gender	−0.06	−0.39	0.695	−0.036
Over75	0.40	−2.42	0.017*	−0.218
Education level	0.47	4.55	<0.001***	0.387
Work	−0.02	−0.21	0.833	−0.019
Domicile	−0.07	−0.40	0.690	−0.036
Marital status	−0.10	−0.53	0.591	−0.049

Variables	Visuo-spatial abilities total score			
	*β*	*t*	*p*	Partial correlation
Gender	0.25	1.39	0.164	−0.036
Over75	−0.19	−1.06	0.289	−0.218
Education level	0.35	3.14	0.002**	0.387
Work	−0.00	−0.01	0.987	−0.019
Domicile	−0.23	−1.25	0.211	−0.036
Marital status	−0.07	−0.38	0.698	−0.049

*p < 0.5, **p < 0.01, ***p < 0.001.

**Table 2:** Multiple regression of subtest of ACE-III’ domains.

Variable	Attention domain			
	*β*	*t*	*p*	Partial correlation
Education level	0.27	2.78	0.006**	0.248
Registration of 3 items				
Education level	0.24	2.43	0.016*	0.219
Subtractions				
Education level	0.28	2.92	0.004**	0.261
Domicile	−0.18	−2.19	0.030*	−0.198
Memory domain				
Learning				
Education level	0.54	5.24	<0.001***	0.436
Semantic memory				
Education level	0.33	2.97	0.003**	0.264
Long-term memory				
Education level	0.22	1.99	0.048*	0.182
Recognition				
Education level	0.28	2.46	0.015*	0.223
Fluency domain				
Verbal fluency				
Education level	0.40	3.97	<0.001***	0.344
Animal fluency				
Education level	0.24	2.13	0.035*	0.193
Language domain				
Language comprehension				
Education level	0.28	2.48	0.014*	0.223
Writing				
Education level	0.23	2.07	0.040*	0.190
Words repetition				
Education level	0.26	2.32	0.0216*	0.210
Phrases repetition				
Education level	0.34	2.99	0.003**	0.266
Domicile	0.42	2.18	0.030*	0.198
Denomination				
over75	−0.42	−2.35	0.020*	−0.213
Education level	0.34	3.06	0.002**	0.273
Comprehension of denomination task				
Education level	0.45	4.34	<0.001***	0.373
Reading				
Education level	0.53	4.85	<0.001***	0.412
Work	−0.23	−2.18	0.031*	−0.199
Visuo-spatial’s ability				
Intersecting infinity loops copying				
Over75	−0.49	−2.78	0.006**	−0.250
Education level	0.22	2.00	0.047*	0.183
Cube copying				
Education level	0.44	4.17	<0.001***	0.361
Recognition of fragmented letters				
Education level	0.28	2.39	0.018*	0.217

*p < 0.5, **p < 0.01, ***p < 0.001.

### 3.1 Gender

Across all cognitive domains, gender did not emerge as a meaningful predictor of performance. Estimates were non-significant and accompanied by negligible partial correlations, indicating that gender-related differences in attention, memory, fluency, language, and visuo-spatial abilities were statistically absent. This pattern suggests that cognitive variability in this sample is better explained by other sociodemographic factors rather than by gender.

### 3.2 Age over 75

Age over 75 showed limited but specific associations with cognitive performance.

In the total scores, age did not significantly predict attention (*β* = −0.02, *p* = 0.887), memory (*β* = −0.25, *p* = 0.145), fluency (*β* = −0.11, *p* = 0.495), or visuospatial abilities (*β* = −0.19, *p* = 0.289).

The influence of being over 75 years old was selective and domain-specific. In the language domain, older participants showed significantly lower total language scores (*β* = 0.40, *t* = −2.42, *p* = 0.017, partial *r* = −0.21). At the subtest level, age significantly predicted denomination (*β* = −0.42, *p* = 0.020, partial *r* = −0. 21) and intersecting infinity loops copying (*β* = −0.49, *p* = 0.006, partial *r* = −0.25). No other subcomponents showed significant age effects.

### 3.3 Education level

Education level was significantly associated with performance across all total cognitive scores. It predicted attention (*β* = 0.36, *p* = 0.001, partial *r* = 0.28), memory (*R*^2^ = 0.141, *p* = 0.006, *β* = 0.44, *p* < 0.001, partial *r* = 0. 35), fluency (*β* = 0.37, *p* < 0.001, partial *r* = 0.31), language (*β* = 0.47, *p* < 0.001, partial *r* = 0.38), and visuospatial abilities (*β* = 0.35, *p* = 0.002, partial *r* = 0.38). Education also showed significant effects across all subtests. In attention, it predicted spatial orientation (*β* = 0.27, *p* = 0.006, partial *r* = 0.24), registration of 3 items (*β* = 0.24, *p* = 0.016, partial *r* = 0.21), and subtractions (*β* = 0.28, *p* = 0.004, partial *r* = 0.26). In memory, it predicted learning (*β* = 0.54, *p* < 0.001, partial *r* = 0.43), semantic memory (*β* = 0.33, *p* = 0.003, partial *r* = 0.26), long-term memory (*β* = 0.22, *p* = 0.048, partial *r* = 0.18), and recognition (*β* = 0.28, *p* = 0.015, partial *r* = 0.22). The model for fluency explained a substantial proportion of variance (*R*^2^ = 0.255, *F*(6,117) = 6.67, *p* < 0.001), with education showing a robust effect in verbal fluency (*β* = 0.40, *p* < 0.001, partial *r* = 0.34) and also in animal fluency (*β* = 0.24, *p* = 0.035, partial *r* = 0.19). In language, it predicted comprehension (*β* = 0.28, *p* = 0.014, partial *r* = 0.22), writing (*β* = 0.23, *p* = 0.040, partial *r* = 0.19), word repetition (*β* = 0.26, *p* = 0.022, partial *r* = 0.21), phrase repetition (*β* = 0.34, *p* = 0.003, partial *r* = 0.26), denomination (*β* = 0.34, *p* = 0.002, partial *r* = 0.27), comprehension of denomination task (*β* = 0.45, *p* < 0.001, partial *r*=0. 37), and reading (*β* = 0.53, *p* < 0.001, partial *r* = 0.41).

In visuospatial abilities, it predicted infinity loops copying (*β* = 0.22, *p* = 0.047, partial *r* = 0.18), cube copying (*β* = 0.44, *p* < 0.001, partial *r* = 0.36), and fragmented letters recognition (*β* = 0.28, *p* = 0.018, partial *r* = 0.21).

### 3.4 Work status

Work status did not significantly predict performance in most cognitive domains. A single exception emerged in reading, where individuals not currently working showed slightly lower performance (*β* = −0.23, *p* = 0.031, partial *r* = −0.19). No other subcomponents showed significant associations with work status.

### 3.5 Domicile

Domicile showed limited associations with cognitive performance. In the total scores, domicile was not a significant predictor for attention, memory, fluency, language, or visuospatial abilities. At the subtest level, domicile significantly predicted subtractions (*β* = −0.18, *p* = 0.030, partial *r* = −0.19) and phrase repetition (*β* = 0.42, *p* = 0.030, partial *r* = 0.19). No other subtests showed significant effects.

### 3.6 Marital status

Marital status did not significantly predict performance in any cognitive domain. Across attention, memory, fluency, language, and visuospatial abilities, all effects were non-significant with negligible partial correlations.

## 4 Discussion

Dementia is a neurodegenerative, progressive, chronic disease that is emerging as one of the most urgent global health challenges, with cases increasing exponentially worldwide. The increasing prevalence of dementia has led to a growing focus on the importance of early diagnosis, which enables timely treatment, improves disease management and, in some cases, slows the progression of symptoms. Within this framework, understanding how sociodemographic factors influence cognitive performance can help to create more effective and targeted screening strategies. The present study investigated the relationships between sociodemographic factors, including gender, age, level of education, type of profession, place of residence, and marital status, and cognitive domines.

The analysis of cognitive performance across the ACE-III domains showed that gender did not contribute to variability in any cognitive function. This result is fully consistent with previous evidence, including the Italian validation of the ACE-III (Pigliautile et al., 2019), which similarly reported no gender differences in global or domain-specific scores. The absence of gender effects suggests that, in adulthood, cognitive performance is shaped primarily by other sociodemographic characteristics. Age showed a more nuanced pattern. Contrary to the generalized decline often described in the literature (Harada et al., 2013), being over 75 did not significantly affect total scores in attention, memory, fluency, or visuospatial abilities. Instead, age exerted selective effects, emerging only in the language domain and in specific subtests. Older adults showed lower performance in denomination and in the copying of intersecting infinity loops, tasks that rely on lexical retrieval and fine visuospatial integration. These findings align with studies indicating that certain cognitive processes, particularly temporal and spatial orientation, lexical access, and complex visuospatial integration, are sensitive to aging (Zancada-Menendez et al., 2016; Serino et al., 2018). Age-related changes in hippocampal functioning may also contribute to these difficulties, given its role in temporal processing and memory for elapsed time (El Haj and Kapogiannis, 2016).

Among all sociodemographic variables, education emerged as the most robust predictor of cognitive performance. Higher educational attainment was associated with better scores in every ACE-III domain and in all subtests, confirming the strong link between education and cognitive functioning reported in previous studies (Weiss et al., 2020; Aki̇ et al., 2022; Olmos-Villaseñor et al., 2023). This widespread effect supports the cognitive reserve hypothesis, according to which education enhances neural efficiency and compensatory mechanisms across the lifespan. The particularly strong associations observed in memory, fluency, language, and visuospatial tasks are consistent with the idea that education contributes to the development of flexible cognitive strategies and richer semantic networks. Work status showed a very limited contribution to cognitive performance. The only significant effect emerged in reading, where individuals not currently working obtained slightly lower scores. This isolated association may reflect the cognitive stimulation provided by occupational engagement, but the overall pattern suggests that work status plays a secondary role compared to education. This interpretation is consistent with the literature on cognitive reserve, which highlights the cumulative contribution of education, life experiences, and intellectually demanding activities (Oosterhuis et al., 2023; Stine-Morrow and Manavbasi, 2022). Recent findings also indicate that cognitive reserve particularly benefits language-related abilities, with stronger effects in fluency tasks (Feldberg et al., 2024), a pattern compatible with the present results.

Domicile also showed limited associations with cognitive performance. While total domain scores were not influenced by living in urban versus rural areas, two subtests, subtractions and phrase repetition, showed significant differences. Although these effects were modest, they may reflect contextual or environmental influences on tasks requiring sustained attention or linguistic integration. Previous research suggests that rural or suburban environments may promote healthier lifestyles, lower stress levels, and greater social interaction (Jimenez et al., 2021), whereas urban settings may expose individuals to chronic stressors that negatively affect cognitive aging (Belsky and Baccarelli, 2023). Environmental quality and exposure to stressors have also been linked to biological aging processes, including epigenetic modifications (Ferrucci et al., 2024). As highlighted by Crimmins (2020), understanding aging requires integrating both social and biological mechanisms, which collectively shape health trajectories across the lifespan. Finally, marital status did not show any association with cognitive performance. All effects were non-significant with negligible correlations, suggesting that, in this sample, marital status does not meaningfully contribute to cognitive variability once other demographic factors are considered.

Overall, the results of this study indicate that among the sociodemographic characteristics examined, education plays the most substantial role in shaping cognitive performance, while age exerts selective effects and other variables show only minor or isolated associations. Promoting higher education, facilitating access to cognitively stimulating activities, and fostering supportive living environments may therefore represent effective strategies for maintaining cognitive health and preventing dementia. Identifying individuals at risk based on sociodemographic characteristics could also support the development of targeted interventions, improving quality of life and reducing the societal burden associated with dementia.

The main limitation of this study is the use of convenience sample in the southern part of Italy, which may limit the generalizability of the findings. This geographic and demographic area could introduce regional biases related to socioeconomic status or educational background, or attitude toward aging and cognitive health.

## 5 Conclusion

Dementia is a global emergency, as evidenced by the alarming epidemiological data. The neurodegenerative, progressive, and chronic nature of the disease makes it a difficult condition to manage at the medical, psychological, and socioeconomic levels. The scientific literature is shifting towards the detection of risk factors for the purpose of early diagnosis. The characteristics of specific populations are essential for developing effective strategies for the prevention, early detection, and management of dementia at both the individual and social levels. This study aimed to investigate socio-demographic risk factors in a sample of people with dementia. The results showed an association between age, education, manual work, living in urban areas and cognitive performances. The results indicate that these variables should be taken into account in diagnostic procedures.

## Data Availability

The raw data supporting the conclusions of this article will be made available by the authors, without undue reservation.
